# Investigating the sub-regions of the superior parietal cortex using functional magnetic resonance imaging connectivity

**DOI:** 10.1186/s13244-021-00993-9

**Published:** 2021-04-13

**Authors:** Adnan A. S. Alahmadi

**Affiliations:** Department of Diagnostic Radiology, College of Applied Medical Science, King Abdulaziz University , Jeddah, Saudi Arabia

**Keywords:** FMRI, Functional connectivity, Superior parietal cortex

## Abstract

**Objectives:**

Traditionally, the superior parietal lobule (SPL) is usually investigated as one region of interest, particularly in functional magnetic resonance imaging (fMRI) studies. However, cytoarchitectonic analysis has shown that the SPL has a complex, heterogeneous topology that comprises more than seven sub-regions. Since previous studies have shown how the SPL is significantly involved in different neurological functions—such as visuomotor, cognitive, sensory, higher order, working memory and attention—this study aims to investigate whether these cytoarchitecturally different sub-regions have different functional connectivity to different functional brain networks.

**Methods:**

This study examined 198 healthy subjects using resting-state fMRI and investigated the functional connectivity of seven sub-regions of the SPL to eight regional functional networks.

**Results:**

The findings showed that most of the seven sub-regions were functionally connected to these targeted networks and that there are differences between these sub-regions and their functional connectivity patterns. The most consistent functional connectivity was observed with the visual and attention networks. There were also clear functional differences between Brodmann area (BA) 5 and BA7. BA5, with its three sub-regions, had strong functional connectivity to both the sensorimotor and salience networks.

**Conclusion:**

These findings have enhanced our understanding of the functional organisations of the complexity of the SPL and its varied topology and also provide clear evidence of the functional patterns and involvements of the SPL in major brain functions.

## Key points

- The SPL has a complex, heterogeneous topology that comprises more than seven sub-regions
- The functional connectivity of these sub-regions to different functional brain networks was investigated.
- There are similarities and differences between these sub-regions and their functional connectivity patterns.
- The most consistent functional connectivity was observed with the visual and attention networks.
- There were also clear functional differences between Brodmann area (BA) 5 and BA7.

## Introduction

The superior parietal lobule (SPL) plays an important role in different brain functions including visuomotor, cognitive, sensory, higher order, working memory and attentional [1–10]. Most of these findings were investigated using functional magnetic resonance imaging (fMRI) based on specific experimental tasks, and the findings reported relate to the SPL as a single region.

Another powerful and useful technique that aids the understanding of the brain’s functional networking mechanism is resting-state fMRI (rsfMRI), which helps to determine the functional integrations of regions of the brain during rest [11]. rsfMRI is also a powerful tool regarding the structural understanding of cortical organisations, as emerging evidence from studies have shown that these functional, spontaneous low-frequency signals were significantly structurally correlated [12–14]. Evidence also suggests that these spontaneous fluctuations observed during rsfMRI produced functional networks that were continuously correlated with experimental tasks [13].

In addition, and in an effort to further understand the cortical organisation of the SPL, recent studies used cytoarchitecture analysis and probabilistic maps in ten post-mortem brains to analyse the SPL [15–17]. The studies showed that the SPL was sub-divided into eight sub-regions: 5 Ci, 5 M, 5L, 7PC, 7A, 7P, 7 M and hIP3. Three of these sub-regions are located in Brodmann (BA) 5, and four of them are located in BA7. Each of these sub-regions is different based on the receptor distribution patterns and the regional cytoarchitectonic properties.

The functional roles of each of these sub-regions have still not been fully investigated, and most of the previous studies mentioned above claimed the involvement of SPL as a single cortical region. The assumption is that since these sub-regions are different from an anatomical point of view, and each has different cytoarchitectonic properties, their differences can expect to be observed during functional connectivity to major network functions. Therefore, this study aims to use a large cohort of subjects to investigate the functional connectivity of each of these seven sub-regions of the SPL with major functional identified networks and to use rsfMRI to determine how these sub-regions differ based on functional connectivity.

## Methods

### Subject recruitments and scanning

A total of 198 healthy volunteer subjects were recruited for this study. Their ages ranged from 18 to 30, and 123 of the subjects were female. 171 of the subjects were right handed. The data were collected and downloaded from the Cambridge-Buckner dataset, which is part of the 1,000 Functional Connectomes Project (an open-access platform without any restrictions, https://www.nitrc.org/projects/fcon_1000/) [12]. This is a non-restricted public dataset and is available to all. The IRP statement provided on the 1,000 Functional Connectomes Project website is shown on the following paragraph.

“The 1,000 Connectomes Project data-sharing effort will provide the research community with open access to datasets contributed by labs around the world. Datasets provided to the 1,000 Connectomes Project are to be de-identified prior to deposition of the data with the project (i.e. removal of any personal identifying information from header/support files). Upon arrival, datasets are automatically organised, and header files are replaced with novel header files to guarantee that any identifying personal information within the header or supporting files is removed. Prior to open-access sharing via web-based repository, all datasets will be further de-identified and anonymised by the removal of face information from the image to prevent any inappropriate reconstruction of the image that could lead to the identification of a participant. Furthermore, each participant's dataset will be assigned a randomised five-digit participant identifier, along with a site identifier (two letters which map to the site providing the data). The relationship between the anonymised code and the original subject ID will be destroyed to ensure that the dataset will be truly anonymised. For each dataset, demographic information provided via web-archive will be limited to (when available): age (18 and up), gender (male, female) and handedness. This information will serve to facilitate more careful characterisation of the data, without entailing a risk of violation of confidentiality. Datasets will only be included in the repository upon receipt of written expressed permission for usage of the dataset freely by the general public, without limitation.”

This dataset was acquired using a typical rsfMRI protocol that is used by previous studies, see for review and references [18–20]. The registered clinical name for this project is the Cambridge-Buckner dataset. All of the 198 subjects were scanned using a Siemens 3-T Trim Trio scanner with the following parameters: T2* weighted Echo Planner Imaging (EPI) sequence with repetition time (TR) = 3 s; TE = 30 ms; number of slices = 47 interleaved axial slices; voxel size: 3.0 × 3.0 × 3.0 mm^3^; number of time points (i.e. volumes) = 124 volumes (the first 5 volumes were discarded). T1-weighted MPRAGE images with the following parameters: number of slices: 192; matrix size 144 × 192; voxel size: 1.20 × 1.00 × 1.33 mm^3^.

### Pre-processing

The pre-processing and statistical analyses were performed using CONN and Statistical Parametric Mapping software (SPM12). The pre-processing of the rsfMRI imaging volumes included such typical pre-processing steps as slice timing corrections, realignment of the functional volumes, normalisation of the functional volumes to an MNI template using structural data, detection data outliers using the implanted artefact detection tools (ART) in CONN [21], and smoothing the functional volumes using an 8-mm kernel. In addition, in order to neutralise the effects of artefacts and confounds (e.g. white matter, CSF signals, and motion and scrubbing parameters) from the BOLD signal, temporal processing with data denoising was also applied.

### Selection of the regions of interest

**Fig. 1:**
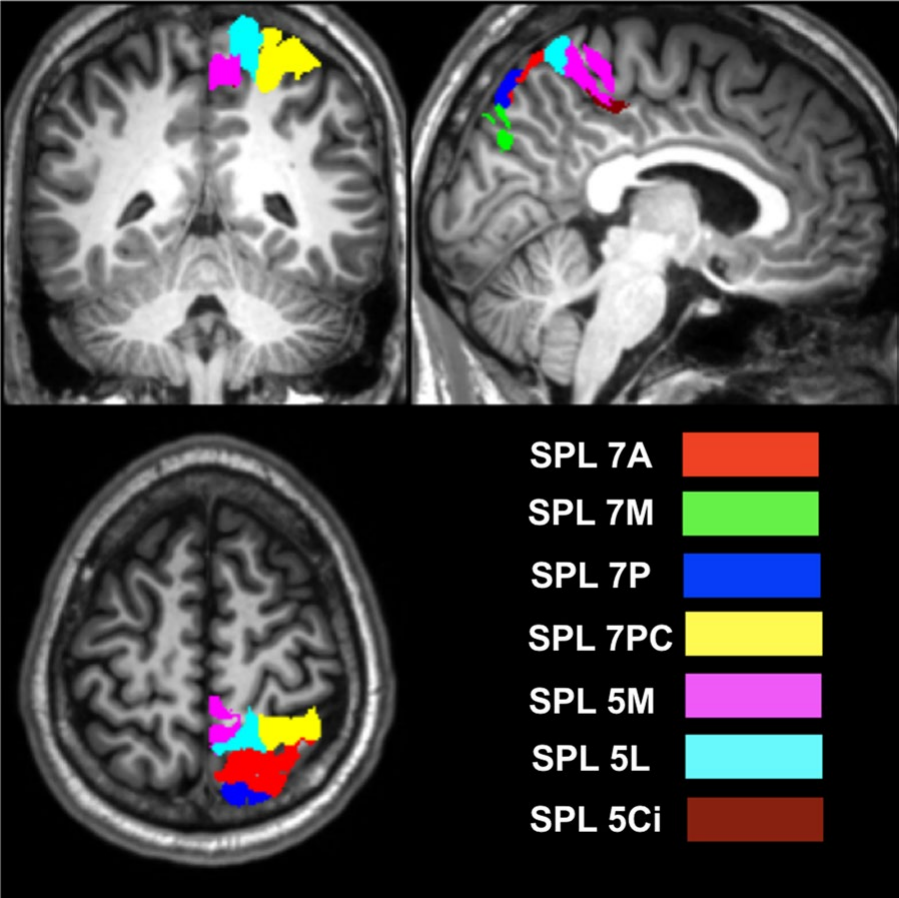
Illustrations of the seven SPL source regions used in this study in the right hemisphere

The seeds identified in this study were the seven sub-regions of the SPL, namely the BA5 sub-regions (5 Ci, 5 M, and 5L) and the BA7 sub-regions (7PC, 7A, 7P, and 7 M) (Fig. 1). These regions were identified using the cytoarchitectonic probability anatomy map, which is guided by the cytoarchitectonic properties of these regions based on the data from ten post-mortem brains [15, 17, 22, 23]. The target brain regions were major collections of functional networks, as defined in CONN and shown in Table 1. In addition to the default mode, the functional networks studied were attention, sensorimotor, visual, salience, dorsal attention, frontoparietal, cerebellar, and language. This atlas of commonly used networks was defined based on an independent component analysis (ICA) of the human connectivity project using 497 subjects.

**Table 1:** Targeted networks with the regions that comprise them. Also shown the coordinates of these regions

Network	Regions
Default mode	MPFC (1,55,−3)
	LP (L) (−39,−77,33)
	LP (R) (47,−67,29)
	PCC (1,−61,38)
Sensorimotor	Lateral (L) (−55,−12,29)
	Lateral (R) (56,−10,29)
	Superior (0,−31,67)
Visual	Medial (2,−79,12)
	Occipital (0,−93,−4)
	Lateral (L) (−37,−79,10)
	Lateral (R) (38,−72,13)
Salience	ACC (0,22,35)
	Insula (L) (−44,13,1)
	Insula (R) (47,14,0)
	RPFC (L) (−32,45,27)
	RPFC (R) (32,46,27)
	SMG (L) (−60,−39,31)
	SMG (R) (62,−35,32)
Dorsal Attention	FEF (L) (−27,−9,64)
	FEF (R) (30,−6,64)
	IPS (L) (−39,−43,52)
	IPS (R) (39,−42,54)
Fronto-Parietal	LPFC (L) (−43,33,28)
	PPC (L) (−46,−58,49)
	LPFC (R) (41,38,30)
	PPC (R) (52,−52,45)
Language	IFG (L) (−51,26,2)
	IFG (R) (54,28,1)
	pSTG (L) (−57,−47,15)
	pSTG (R) (59,−42,13)
Cerebellar	Anterior (0,−63,−30)
	Posterior (0,−79,−32)

Abbreviation: MPFC: medial prefrontal cortex; LP: lateral parietal; PCC: posterior cingulate cortex; ACC: anterior cingulate cortex; RPFC: rostrolateral prefrontal cortex; SMG: supramarginal gyrus; FEF: frontal eye fields; IPS: intraparietal sulcus; LPFC: lateral prefrontal cortex; PPC: posterior parietal cortex; LPFC: lateral prefrontal cortex; IFG: inferior frontal gyrus; pSTG: posterior superior temporal gyrus

### Statistical analysis

Statistical analysis was calculated at two levels. At the first level (subject-level), weighted general linear bivariate correlation models, including regions of interests (ROI)-to-ROI connectivity matrices between the pre-defined ROIs, were calculated for each subject. These connectivity matrices were defined as Fisher-transformed bivariate correlation coefficient between the pair of ROI timeseries. At the second level, functional connectivity measures were calculated and compared using group-level statistical analysis, such as T-tests and/or F-tests where appropriate, and identifying and comparing the rsfMRI networks connected to each of the sub-regions of the SPC at the group level. The standard setting for the results is displayed using a corrected false discovery rate (FDR) (p < 0.05) (multivariate statistics parametric (MVPA) omnibus test)[21]. Here, cluster-level inferences are determined using functional network connectivity multivariate parametric statistic inferences by considering groups or networks of related ROIs. Then, the analysis is done by analysing the entire set of connections between the ROIs in terms of within- and between-network connectivity [24]. This method effectively performs a multivariate parametric general linear model analysis for all connections. The resultant map is of F-statistical tests for each pair of networks. The FDR cluster level is defined as the expected proportion of false discoveries among all pairs of networks with similar or larger effects across the entire set of functional connectivity network pairs [25]. One of the reasons for using the FDR over family wise error (FWE) is that the FDR is more sensitive in controlling peaks, with minimal cost of false positives [26]. For additional details, please refer to the above papers or the CONN website (https://web.conn-toolbox.org).

## Results

**Fig. 2:**
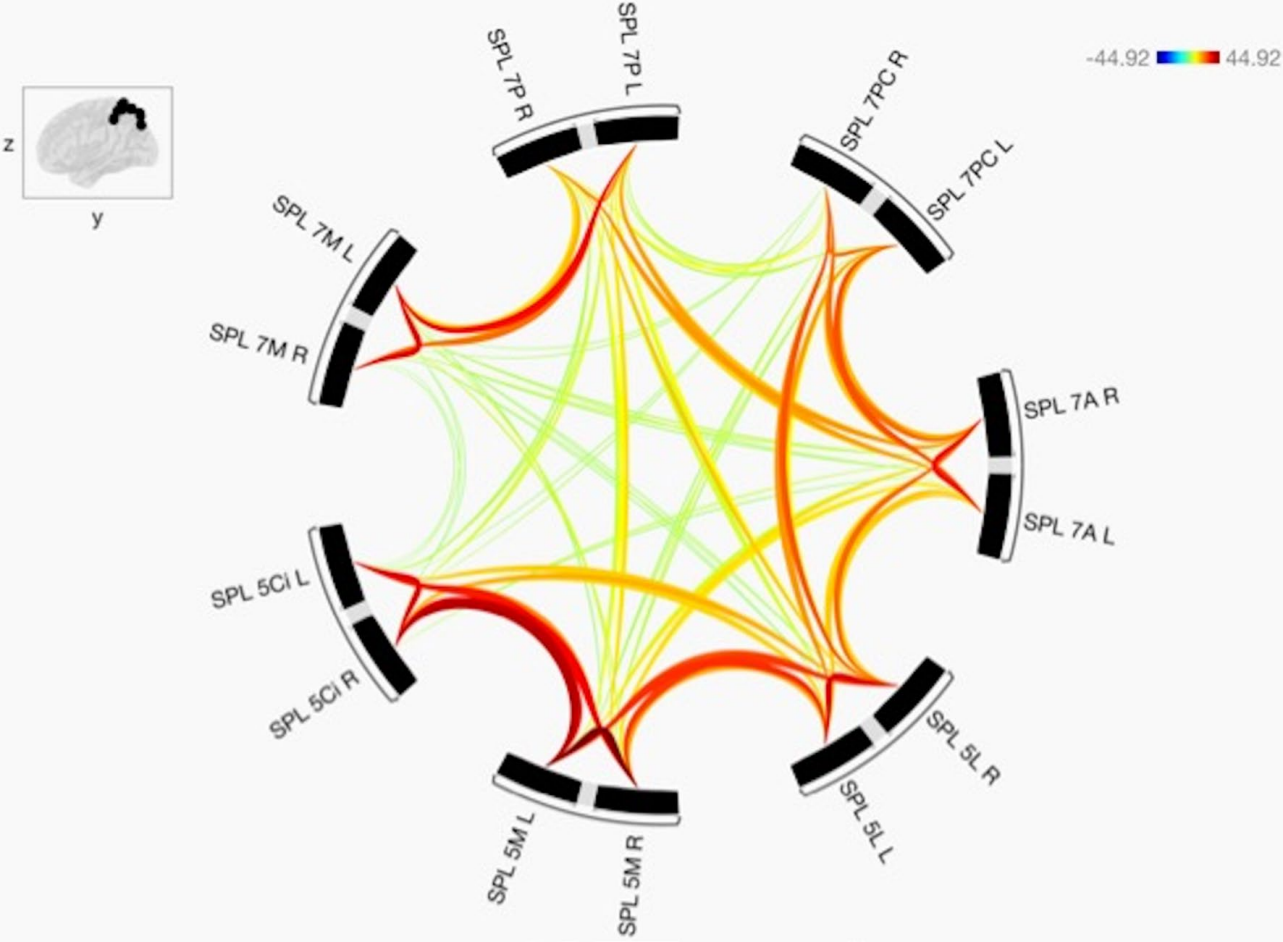
The functional connectivity among the sub-regions of the SPL is shown. These are the seven investigated sub-regions of the SPL in right and left hemispheres (14 sub-regions). The lines of the connections in red indicate positive connectivity, and these colours are proportional to statistical strength. Additionally, the T-bar is shown in the top right-hand corner

All of the sub-regions are connected to each other, and the functional connectivity between the seven sub-regions of the SPL is shown in Fig. 2. Figure 2 also shows that the strength of each of the connections is statistically different from each other.

**Fig. 3:**
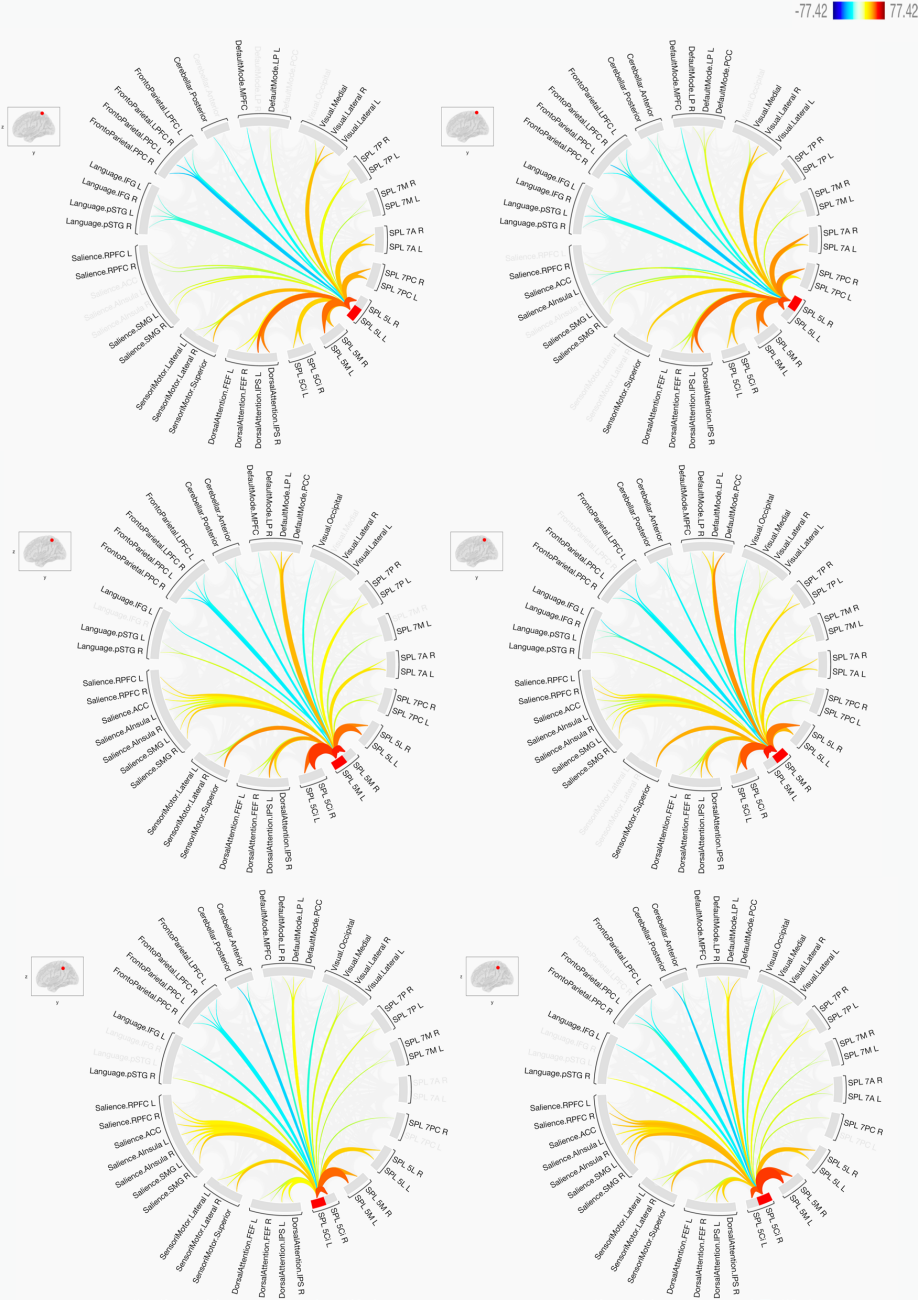
The functional connectivity of the three SPL sub-regions of BA5 in both hemispheres is shown here. The lines of the connections in red indicate positive connectivity while blue indicates negative connectivity. The colours of the lines are proportional to statistical strength, and the T-bar is shown in the top right-hand corner

**Fig. 4:**
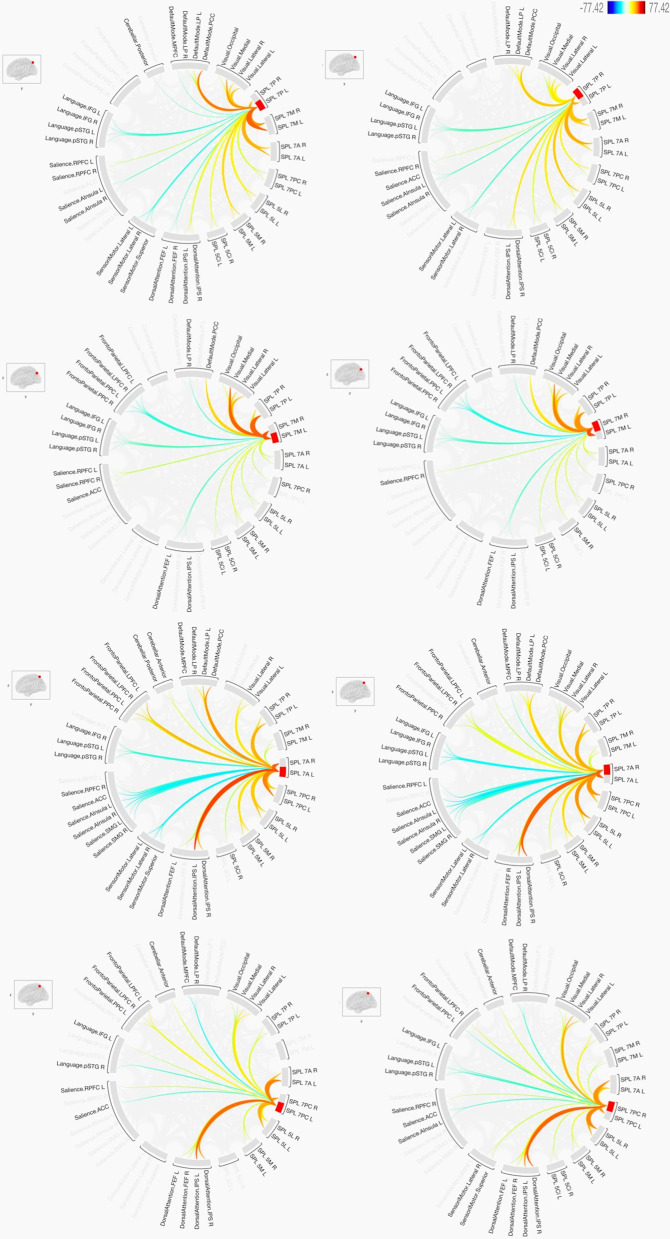
The functional connectivity of the four SPL sub-regions of BA 7 in both hemispheres is shown here. The lines of the connections in red indicate positive connectivity while blue indicates negative connectivity. The colours of the lines are proportional to statistical strength, and the T-bar is shown in the top right-hand corner

**Fig. 5:**
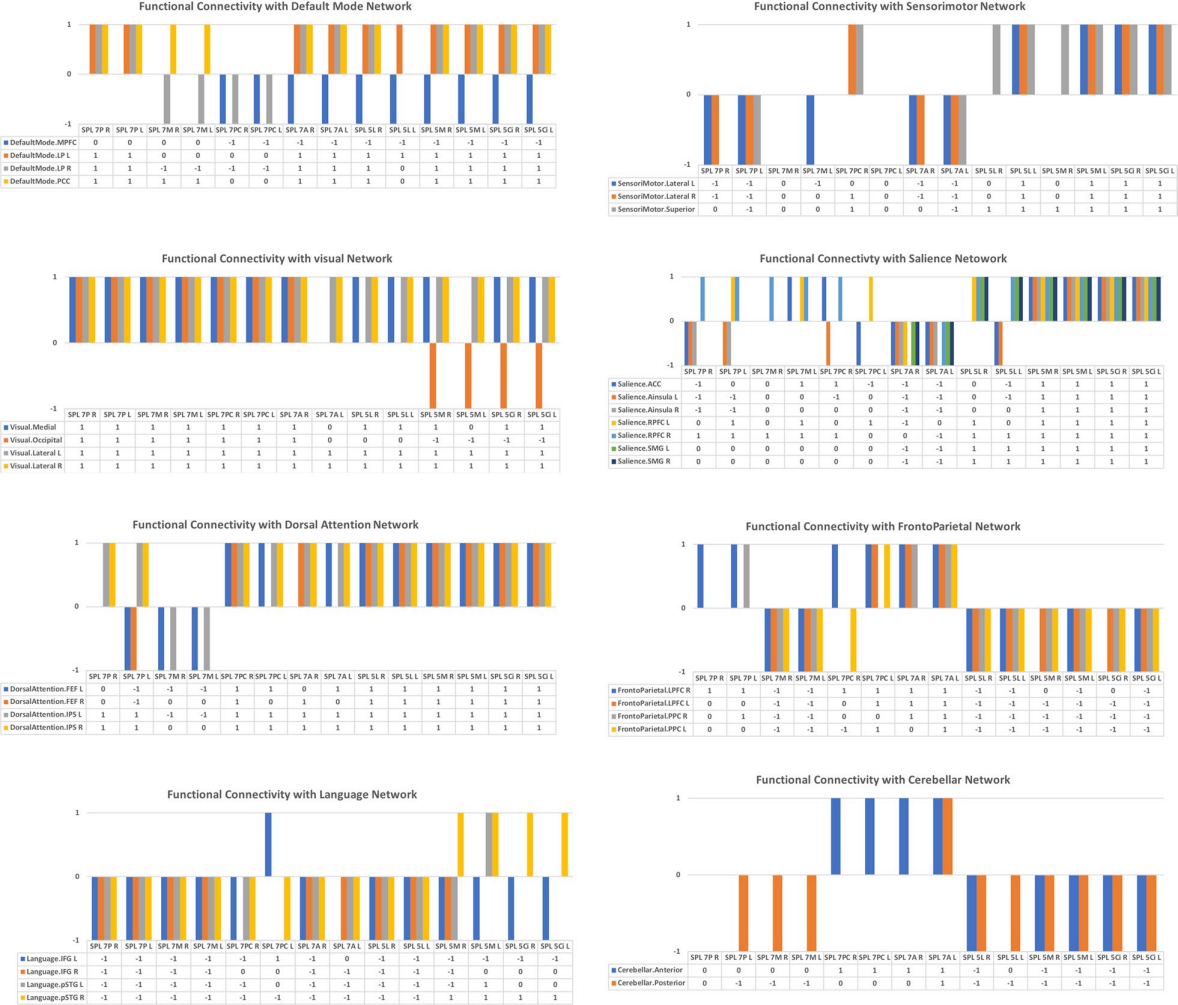
The functional connectivity of all the SPL sub-regions in both hemispheres is shown here, along with showing whether each sub-region is connected positively (+ 1), is not connected (0) or is connected negatively (-1) with the eight targeted networks

The study investigated the functional connectivity between the seven sub-regions of the SPC in the two hemispheres of the brain to the eight targeted networks. In general terms, the results showed that there are strong connections (either positive or negative) between the sub-regions and the targeted networks. There were similarities and differences among the connections, which are summarised in Figs. 3 and 4. Additionally, Fig. 5 summarises the connections by indicating whether there is a positive connection, a negative connection, or no connection. The key findings are shown below.

### Functional connectivity with the default mode network

In general, the seven sub-regions of the SPL had positive connections with the regions defining the default mode network, particularly the lateral parietal (LP) and posterior cingulate cortex (PCC). Negative connections were also observed, particularly with the medial prefrontal cortex (MPFC) and within BA5. The results also indicated that there were no significant differences between the two hemispheres as most of the connections to these sub-regions of the SPL were identical. The sub-regions that had clear and distinct differences from the other sub-regions were the SPL 7 M, the SPL 7PC and the left SPL 5L.

### Functional connectivity with the sensorimotor network

In general, most of the sub-regions of the SPL located within BA7 had negative connections with the sub-regions defining the sensorimotor network, while all the sub-regions of the SPL located within BA5 had positive connections. In contrast, the right SPL 7 M and the left SPL 7PC had no connections at all.

### Functional connectivity with the visual network

In general, most of the sub-regions of the SPL had positive connections with the regions defining the visual network. This was highly constant within the sub-regions of BA7. Within the sub-regions of BA5, there were negative connections with the SPL 5 M and SPL 5 Ci.

### Functional connectivity with the salience network

There were clear differences between the sub-regions of the SPL within BA7 and the regions defining the salience network. These were either defined by positive, negative or no connections at all. While the sub-regions of SPL within BA5 had mostly positive connections, the sub-regions of the SPL within BA7 contained differences between the two hemispheres when functional connectivity with the salience network was investigated. The largest number of positive connections of the SPL BA7 sub-regions were seen with the rostrolateral prefrontal cortex (RPFC) of the salience network, while the SPL 7A had negative connections with most of the regions making up the salience network.

### Functional connectivity with the dorsal attention network

In general, most of the sub-regions of the SPL had positive connections with the regions defining the dorsal attention network. This was highly constant within the sub-regions of BA5. Within the sub-regions of BA7, there were negative connections with the SPL 7 M and left SPL 5P. SPL 7P only had positive connections with the intraparietal sulcus (IPS) of the dorsal attention network, while the left SPL 7P had negative connections with the frontal eye fields (FEF) of the dorsal attention network. The SPL 7 M had negative connections in both hemispheres with the FEF and IPS of the dorsal attention network, while it had no connections with the IPS.

### Functional connectivity with the frontoparietal network

In general, most of the sub-regions of the SPL had negative connections with the regions making up the frontoparietal network. These negative connections were constant within the sub-regions of BA5. The sub-regions of BA7 were heterogeneous in terms of their functional connections with the regions defining the frontoparietal network, while SPL 7 M had negative connections. Positive connections were only observed in SPL 7P with the right lateral prefrontal cortex (LPFC) and the posterior parietal cortex (PPC) of the frontoparietal network. Hemispheric differences were observed in SPL 7PC, with the right SPL 7PC having a negative connection with the left PPC of the frontoparietal network.

### Functional connectivity with the language network

In general, most of the sub-regions of the SPL had negative connections with the regions defining the language network. These negative connections were mostly within the sub-regions of BA7. The only region that had a positive connection with the language network within BA7 was the left SPL 7PC, which had a positive connection with the left inferior frontal gyrus (IFG). The sub-regions of BA5 were partially heterogeneous in terms of their functional connections with the regions defining the language network. Two sub-regions of BA5—SPL 5 M and SPL 5 Ci—had positive connections with the right posterior superior temporal gyrus (pSTG) of the language network. In contrast, the only negative connections in these two sub-regions were with the left IFG. Hemispheric differences were not significantly observed among all of the sub-regions and it was mainly related to the SPL 5 M and SPL 5 Ci.

### Functional connectivity with the cerebellar network

There is a clear difference between the sub-regions of SPL within BA7 and the regions defining the cerebellar network, with heterogeneous functional connectivity within the BA7 sub-regions being observed. Negative connections with the posterior cerebellum were constant with all the sub-regions of BA5, while negative connections were also observed with the SPL 7 M and the left SPL 7P. The anterior cerebellum also showed negative connections with most of the sub-regions of BA5. Positive connections were mainly observed between the anterior cerebellum and the SPL 7PC and SPL 7A.

## Discussion

This study used a large dataset to investigate the functional connectivity of seven sub-regions of the SPL with eight regional network functions using rsfMRI. These seven sub-regions were shown to be different from each other both structurally and cytoarchitecturally [15–17, 23, 27]. The assumption was that since these sub-regions were cytoarchitecturally different, their functional connectivity could also be different. In general terms, the findings of this study showed that these sub-regions were connected to the eight functional networks. The functional connectivity was similar between some networks and different between other networks.

The findings showed the important functional role that the SPL plays in terms of its involvements in different functional domains, particularly regarding the attentional and visual pathways. Here, the functional connectivity was most strongly connected with the networks in most of the sub-regions of the SPL. This is in line with previous findings that showed a strong involvement of the SPL with visual and attentional functions [2, 8, 28–30]. However, the connections to the visual and attention networks were neither homogeneous nor consistent among the sub-regions of the SPL.

The results of this study indicated that all of the sub-regions of BA7 were significantly involved with the visual networks, whereas the sub-regions of BA5 were showing some heterogeneous functional connections. For example, there were no relationships between the sub-regions of BA5 with the visual occipital part of the visual network. This visual occipital part was shown to be mainly involved in the perception of visual shapes [13].

This could indicate that not all of the SPL is involved in visual functions. SPL BA7 plays a greater role in visual processing, while SPL BA5 has limited involvements in visual functions but no direct involvement in the perception of visual shapes. When looking at the attentional network, which consisted of FEF and IPS, all of the sub-regions of SPL BA5 were directly involved in attentional functions to a greater extent than the sub-regions of SPL BA7. There was heterogeneous connectivity within the sub-regions of SPL BA7 where only the right SPL 7PC had full connectivity with all of the sub-regions of the attention network. Some studies have shown that the involvement of SPL in attentions was predominant in the right hemisphere [9], which could indicate that this is only true within SPL BA7 but not within SPL BA5.

In addition, the sensorimotor network involves areas that are related to motor and sensory functions, including the pre- and post-central gyri as well as the supplementary motor areas. Indeed, the SPL plays an important role in sensorimotor tasks, particularly visuomotor tasks or tasks that require certain visuomotor attention [8].

The results of this study showed that there were distinct differences between BA5 and BA7. Most of the sub-regions of BA5 had positive connectivity with the sensorimotor networks, particularly the superior region, whereas the BA7 sub-regions, except for SPL 7PC, had either negative or no connections with the sensorimotor network. Previous studies have shown that BA5 has extensive involvement in sensorimotor functions, while BA7 has less involvement [31, 32]. The results indicated that most of the sub-regions of BA5, apart from the right SPL 5 M, were indeed involved with the sensorimotor network.

The results also showed that the right SPL 7PC is the only sub-region in BA7 that was involved with the sensorimotor network, indicating that there are heterogeneous connections in the sub-regions of BA5 and BA7. However, these heterogeneous connections were only found within one of the sub-regions.

The default mode network is a network of interacting brain regions that include the MPFC, the LP and the PCC. These interacting brain regions are related to each other and usually have distinct patterns compared to other networks [33]. Typically, the default mode network is related to intrinsic changes and it has greater remarkable differences in neurological diseases [34–36]. In our study, the seven sub-regions of the SPL had mostly positive connections with the regions defining the default mode network, particularly with the LP and PCC of the default mode network. These findings indicate the importance of these sub-regions in their connections to the default mode network and indicate how the default mode network may play a role in the brain functions controlled by the SPL. The fact that these sub-regions had more positive connections with the LP and PCC of the default mode network could be because of their involvement in higher-order cognitive and attentional tasks.

However, the results also showed that the default mode network had negative connections with the MPFC. This suggests that the default mode and the seven sub-regions of the SPL have some heterogeneous relationships and that the default mode network sub-regions should be investigated as a heterogeneous network. This is in line with studies that showed that the prefrontal cortical part, which had negative connections with most of the SPL sub-regions, and the PCC, which had positive connections with most of the SPL sub-regions, are distinctly different from each other [37].

Our study also showed that there were sub-regions of the SPL that were distinctly different from the other sub-regions, namely the SPL 7 M, SPL 7PC and the left SPL 5L. Recent studies showed that the SPL 7 M, SPL 7PC and SPL 5L showed distinct functional activations or connectivity during different operational tasks [1, 38], which could explain their distinct patterns of connections with the prefrontal part of the default mode network.

Moreover, the involvement of the SPL’s sub-regions with the other remaining networks—namely the salience, frontoparietal, language and cerebellar networks—were heterogeneous and were not strongly connected with all of the sub-regions. In addition, anticorrelation or negative connectivity was often observed between most of the sub-regions of the SPL with these networks. However, the interpretation of these negative functional connections is beyond the scope of this study, particularly as there is still debate regarding the meaning of these anticorrelation signals in functional connectivity [39]. The results of this study could indicate that heterogeneous functional connectivity is present and that the SPL should not be seen or investigated as a whole region of interest.

Two further observations are worth mentioning and highlighting. SPL 7PC and SPL 7A were the two sub-regions found to be positively connected to the anterior cerebellum, which is known to be involved in motor-related functions [40–46]. This suggests that these two sub-regions could have a direct role in organising the pathways and functional involvements of the anterior cerebellum. Also, recent studies have found that SPL 7PC was mainly involved in execution and motor functions [1], which could explain the direct involvement of this sub-region as a motor pathway with the anterior cerebellum. Finally, this study also showed that the SPL BA5 sub-regions were all involved and correlated positivity with the salience network. The salience network is known to be involved in salience functions and attentions [47], which could also suggest that BA5 has a role in driving the pathways of focused and salience functions.

### Methodological and future considerations

Despite the coherent findings in this study and the clear differences among the SPL sub-regions, there are some potential limitations that should be considered in future studies. Future studies could investigate the question of the differences of the functional connectivity of these sub-regions using high-resolution data (i.e. 1 mm3). This may help to enhance the subtle differences among these sub-regions. Usually, such high-resolution data are acquired using high field scanner strength (i.e. 7 T).

In addition, a potential limitation of this study was the applied smoothing kernel. Although 8 mm3 is a default (and considered typical [48–51]) kernel for such voxel resolution, future studies may consider the effects of smoothing on the connectivity of these sub-regions. However, some recent studies have shown that smoothing kernels could have little effects, especially on rs-fMRI ROI to ROI analysis [52, 53]. Regardless of these studies and since this study’s source regions of interest were adjacent, one could argue that the 8 mm3 smoothing kernel would blur those regions and affect the results. Therefore, a minimal smoothing kernel (or no smoothing) could be tested in future studies to compare and validate the results.

Another limitation of this study is the usage of an open-access dataset. The limitation is related to the information that could be used in the statistical method to investigate, for example, if gender and education level could be confounding factors that may have affected the results. Previous studies have shown functional connectivity based on gender differences [54]; however, such factors need careful data acquisition and planning before starting studies, and performing such was beyond the aim of this study. In addition, handedness in this study was shown to have no effect on the investigated results, when used as a covariate in the GLM. This finding is likely because the number of right-handed subjects was large compared to left-handed subjects, and this study did not aim to investigate the effect of handedness on the results. Thus, future studies could design a study to investigate such effects, which probably could affect the functional connectivity of SPL 7A and 5 M with other brain regions.

In addition, one of this study’s limitations is the analysed dataset’s parameters. For example, this study’s number of time points is not large. Longer scans (and more time points) can increase resting state reliability and improve sensitivity [55, 56]. Therefore, future studies could consider better quality datasets to check the reliability of this study’s findings.

Finally, future studies could investigate structural and functional connectivity among those sub-regions to clarify the unexplained connectivity and better understand the physiological organisations underlying the regions with different networks. For example, it may be that the internal interactions spontaneous patterns could be highly structured [1, 27, 57, 58]. Or these functional connections could not be correlated structurally, which may suggest mediations by indirect structural connections [58]. Also, future studies could investigate effective connectivity and task-related activations of these sub-regions [59]. Therefore, a multimodal approach to analyse these regions is needed. For example, a recent study aimed to identify a convergent organisation of the SPL using structural and functional connectivity [1]. The researchers identified five sub-regions (two anterior and three posterior) in the SPL based on multimodal neuroimaging analyses. They were able to show functional-related differences of the identified regions of the brain that were linked to vision, motor control, perception, memory and attention. The findings of their results are in line with the findings of our study in terms of the different functional involvements of the SPL regions. One could therefore combine the usage of these cytoarchitecture identified sub-regions and with structurally identified approaches to further enhance our understanding of the physiological organisations of these sub-regions.

Therefore, it must also be acknowledged that interpreting the specific connectivity patterns of these sub-regions is difficult to do. This is because, on the one hand, different task experimental fMRI needs to be implemented to test for the involvement of these sub-regions in different brain functions. On the other hand, this study is one of the first that attempts to understand the cortical heterogeneous functional connectivity of these cytoarchitecturally different sub-regions.

However, the findings of this study are important because they provide clear evidence that in addition to the cytoarchitectural differences among these seven SPL sub-regions, there are also functional connectivity differences that should also be considered. Investigating the similarities and differences in functional connectivity among these seven sub-regions of the SPL with important targeted functional brain networks should highlight the importance of using more specified detailed anatomical atlases to investigate these cortical regions in more specific ways. It could also help future studies to focus on the impacts of different neurodegenerative diseases, and how the pathologies of such diseases affect the functional connectivity of these sub-regions. The findings of this study could also enhance our understanding of the functional organisations of the complexity of the SPL and its varied topology and could also assist in reviewing the applied specific task functional activation involvements of these sub-regions.

## Conclusion

This study has shown the similarities and differences in functional connectivity between the seven cytoarchitecturally different sub-regions of the SPL with different functional brain networks. The study has shown how each of the SPL sub-regions plays an important role in visual and attentional functions. The study has also shown the various involvements of the sub-regions of BA7 and BA5 in other brain functions. The heterogeneity of the functional connectivity of these sub-regions has also been proven by this study, which suggests that the SPL has different complex structural and functional topological organisations that should always be considered when investigating the physiology of this important cortical region, or in neurological disease applications.

## Data Availability

All data used in the study are available in this website (https://www.nitrc.org/projects/fcon_1000/).

## Abbreviations

ART: Artefact detection tools
BA: Brodmann area
FDR: False discovery rate
fMRI: Functional magnetic resonance imaging
IPS: Intraparietal sulcus
LP: Lateral parietal
LPFC: Lateral prefrontal cortex
MPFC: Medial prefrontal cortex
PCC: Posterior cingulate cortex
ROI: Regions of interests
RPFC: Rostrolateral prefrontal cortex (RPFC)
rsfMRI: Resting-state fMRI
SPL: Superior parietal lobule
